# The Brief Fear of Negative Evaluation Scale (BFNE): translation and validation study of the Iranian version

**DOI:** 10.1186/1471-244X-9-42

**Published:** 2009-07-09

**Authors:** Azadeh Tavoli, Mahdiyeh Melyani, Maryam Bakhtiari, Gholam Hossein Ghaedi, Ali Montazeri

**Affiliations:** 1 Department of Psychology, Faculty of Humanity Studies, Tarbiat Modares University, Tehran, Iran; 2 Department of Psychology, Shahid Beheshti University of Medical Science, Tehran, Iran; 3 Department of Psychiatry, Faculty of Medicine, Shahed University, Tehran, Iran; 4 Iranian Institute for Health Sciences Research, ACECR, Tehran, Iran

## Abstract

**Background:**

The Brief Fear of Negative Evaluation Scale (BFNE) is a commonly used instrument to measure social anxiety. This study aimed to translate and to test the reliability and validity of the BFNE in Iran.

**Methods:**

The English language version of the BFNE was translated into Persian (Iranian language) and was used in this study. The questionnaire was administered to a consecutive sample of 235 students with (n = 33, clinical group) and without social phobia (n = 202, non-clinical group). In addition to the BFNE, two standard instruments were used to measure social phobia severity: the Social Phobia Inventory (SPIN), and the Social Interaction Anxiety Scale (SIAS). All participants completed a brief background information questionnaire, the SPIN, the SIAS and the BFNE scales. Statistical analysis was performed to test the reliability and validity of the BFNE.

**Results:**

In all 235 students were studied (111 male and 124 female). The mean age for non-clinical group was 22.2 (SD = 2.1) years and for clinical sample it was 22.4 (SD = 1.8) years. Cronbach's alpha coefficient (to test reliability) was acceptable for both non-clinical and clinical samples (α = 0.90 and 0.82 respectively). In addition, 3-week test-retest reliability was performed in non-clinical sample and the intraclass correlation coefficient (ICC) was quite high (ICC = 0.71). Validity as performed using convergent and discriminant validity showed satisfactory results. The questionnaire correlated well with established measures of social phobia such as the SPIN (r = 0.43, p < 0.001) and the SIAS (r = 0.54, p < 0.001). Also the BFNE discriminated well between men and women with and without social phobia in the expected direction. Factor analysis supported a two-factor solution corresponding to positive and reverse-worded items.

**Conclusion:**

This validation study of the Iranian version of BFNE proved that it is an acceptable, reliable and valid measure of social phobia. However, since the scale showed a two-factor structure and this does not confirm to the theoretical basis for the BFNE, thus we suggest the use of the BFNE-II when it becomes available in Iran. The validation study of the BFNE-II is in progress.

## Background

Social phobia is characterized by a fear of negative evaluation within social or performance situations, where the individual is under scrutiny and maybe embarrassed [1]. Social phobia, also known as social anxiety disorder is 'a marked and persistent fear of one or more social or performance situations in which the person is exposed to unfamiliar people or to possible scrutiny by others' [2]. Epidemiological studies have revealed that social anxiety disorder is one of the three most common mental disorders and the most common anxiety disorder in adolescence [3]. The reported rates vary considerably depending on the measures used, populations studied or whether prevalence is based upon clinical diagnosis or individual symptoms of anxiety.

The Brief Fear of Negative Evaluation Scale (BFNE) is a measure of a person's tolerance for the possibility they might be judged disparagingly or hostilely by others [4]. This scale measures fear of negative evaluation from others, hallmark criteria for the diagnosis of social phobia and other disorders, and is relevant to the study of human social behavior in general. With questions derived nearly verbatim from the 30-item Fear of Negative Evaluation (FNE) Scale [5], the 12-item BFNE Scale has the practical advantage of brevity, and has become a frequently used instrument in social anxiety research [6]. Leary was the first one that originally established the psychometric properties of the BFNE Scale among a sample of college students. The BFNE Scale was highly correlated with the 30-item FNE Scale (r = 0.96). Internal consistency (α = 0.96) and three-week test-retest reliability (ICC = 0.75) was high [4].

Since the BFNE scale contains two types of items (8 straightforwardly worded items and 4 reverse-worded items), some researchers recommended that reverse-worded items should be removed from scoring [6,7]. In contrast, in order to maintain the scale sensitivity other researchers suggested instead of removing reverse-worded items these items be reworded. This revised version of the BENF scale is known as the BENF-II [8]. Collins et al. using a revised version of the scale having all items straightforwardly worded, in a clinically anxious Canadian sample, found a modest relationship (r = 0.56) to the social phobia subscale of the fear questionnaire. The scale successfully discriminated social anxious from non-anxious individuals. Reliability in the clinical sample was excellent (α = 0.97) with a test-retest correlation of 0.94 over two weeks [9].

There are several studies that examined the factor structure of the BFNE. Rodebaugh et al. [6] found a two-factor solution corresponding to positive and reverse-worded items best fit the data (n = 1049). More recently, Weeks et al. [7] also found a two-factor solution in a clinically anxious sample. Duck et al. [10] in their study on a community sample supported a two-factor model with factors representing positive and reverse-worded items. However, Rodebaugh et al. argued that this factor structure might not be a reflection of two distinct, underlying constructs but rather an artifact of the wording of the questions. The two factors may represent a single construct assessed by two sets of items that use different methods [6]. Thus, as indicated by Carleton et al. in their recent paper performing confirmatory factor analysis, if we change reverse-worded items to straightforward items, then it would become clear that in fact the BFNE is a unitary factor structure scale that conforms to the theoretical basis for the scale without risking loss of sensitivity from its item removal [11]. To sum up, it seems that at present the BFNE-II is a good alternative form of the BFNE for measuring social phobia.

Since the Brief Fear of Negative Evaluation Scale was not available in Iran, this study aimed to translate the scale and report on its psychometrics properties. However, at the time of the present study the authors were not aware of the BFNE-II; otherwise we should have translate and validated this recent version of the scale.

## Methods

### Translation

The 'forward-backward' procedure was applied to translate the BFNE from English into Persian (Iranian language). Two clinical psychologist translated the questionnaire into Persian and two professional translators backward translated these into English. Then, a provisional version of the Iranian questionnaire was developed and pilot tested and after review by a panel of experts (including the study coordinator, a translator and a member of research team); the final version of the questionnaire was provided.

### Participants and data collection

The final draft of the Iranian version of BFNE was administered to a sample of 202 university students (the non-clinical group) who participated in a large questionnaire-based survey. The samples were selected from students of the various faculties of Shahed University in Tehran, Iran. The questionnaires were administrated while they were attending the lectures. In addition, based on Structured Clinical Interviews for Diagnosis-Version IV [12] a sample of 33 anxious students (the clinical group) were identified by university clinical psychologists and entered into the study. They were referred for treatment to a family health clinic at Mostafa Khomeini Hospital, Iran. All Participants completed a brief background information (age, gender, marital status, year in college) questionnaire, the SPIN, the SIAS and the BFNE scales. Verbal consents obtained from all participants prior to interview. The Ethics Committee of the Shahed University approved the study.

### Measures

#### The Brief Fear of Negative Evaluation Scale (BFNE)

The BFNE measures anxiety associated with perceived negative evaluation. This scale is composed of 12 items describing fearful or worrying cognition. The respondent indicates the extent to which each item describes himself or herself on a Likert scale ranging from 1 'Not at all' to 5 'Extremely'. Eight of the twelve items describe the presence of fear or worrying, while the remaining four items describe the absence of fear or worrying. The factor structure is uncertain with some finding a unitary factor structure [4]; whereas others using a clinical sample have found a two-factor structure with factors characterized by positive and reverse worded items [6,9].

#### The Social Phobia Inventory (SPIN)

this is a measure of social anxiety/distress, fear, physiological symptoms and avoidance of social situations. The SPIN contains 17 items and consists of three subscales: fear, avoidance and physiological symptoms. Each of the 17 items is rated on a scale from 0 to 4: not at all, a little bit, somewhat, very much, and extremely; with higher scores corresponding to greater distress the full- scale score thus ranges from 0 to 68. The authors reported an internal consistency of 0.87 to 0.94 in the social phobia subjects and 0.82 to .090 in control groups, and a test-retest reliability of .89 in the social phobia subjects [13]. Validity of the SPIN as performed using divergent, convergent and construct validity showed satisfactory results [13]. Preliminary results of a recent study indicate good psychometric properties for this scale in an Iranian population [14].

#### The Social Interaction Anxiety Scale (SIAS)

this is an easy and quick instrument to use. It comprises 20 items, each with a 5-point Likert scale for answers. The SIAS and the SPIN are used simultaneously to measure complementary aspects of social phobia. The validation study of the SIAS resulted in a high internal consistency (α = 0.93) and test-retest correlation coefficient above 0.90 [15]. The psychometric properties of the Iranian version of the SIAS are well documented [16].

### Statistical analysis

Descriptive statistics including numbers, proportions, means and standard deviations were used to present data. The internal consistency and reliability were evaluated by Cronbach's alpha Coefficient and the test-retest correlation. For the purpose of the test-retest analysis, the non-clinical group completed the BFNE twice; once at the study commence and once 3 weeks later. Validity of the instrument was assessed using the convergent and discriminant validity [17]. Convergent validity was carried out to demonstrate the extent to which the BFNE correlates with scores derived from the SPIN and the SIAS. It was expected that the BFNE would positively correlate with these measures. Discriminant validity was addressed by examining the ability of the BFNE to differentiate between individuals with and without social phobia. Finally the factor structure of the questionnaire was extracted by performing principal component analysis with varimax rotation. It was hypothesized that two factors would be obtained.

## Results

In all 235 students were studied. The characteristics of the both groups are shown in Table 1. There were no significant differences between the non-clinical and clinical samples.

**Table 1:** Demographic characteristics of the clinical and non-clinical samples.

	**Non-clinical sample** **(n = 202)**	**Clinical sample** **(n = 33)**	**P**
	**No. (%)**	**No. (%)**	

**Age**			0.45

Mean (SD)	22.2 (2.1)	22.4 (1.8)	

Range	19–27	19–25	

**Gender**			0.33

Male	98 (45)	13 (39)	

Female	104 (55)	20 (61)	

**Marital status**			0.24

Single	153 (77)	28 (85)	

Married	49 (23)	5 (15)	

**Year in college**			0.15

First year	89 (44)	14 (43)	

Second year	43 (21)	7 (21)	

Third year	38 (19)	7 (21)	

Forth year	32 (16)	5 (15)	

The internal consistency of the BFNE as assessed by Cronbach's alpha coefficient showed satisfactory results. Cronbach's alpha coefficient was 0.90 for non-clinical group, and was 0.82 for clinical group (social phobic students). In addition, test-retest reliability of the BFNE showed satisfactory results (Intraclass correlation coefficient = 0.71, p < 0.001).

Validity of the BFNE was examined using the convergent analysis. Convergent validity was assessed using the correlation between the BFNE score and the Iranian versions of the SPIN and the SIAS. As expected a significant positive correlation emerged. The results are shown in Table 2.

**Table 2:** The correlation between the BFNE, the SPIN, and the SIAS

	**BFNE**	**SPIN**	**SIAS**
**BFNE**	1		

**SPIN**	0.43*	1	

**SIAS**	0.54*	0.68*	1

* All p values less than < 0.01.

To assess the discriminant validity, the BFNE scores among individuals with and without social phobia were compared. Table 3 displays the results. The scale differentiated well between two groups who differed in social phobia. As hypothesized individuals with social phobia scored lower on the BFNE and other measures and the differences were significant.

**Table 3:** The comparison of the BFNE, the SPIN, and the SIAS scores among clinical and non-clinical samples.

	**Non-clinical sample** **(n = 202)**	**Clinical sample** **(n = 33)**	**Effect size**	**p**
**Fear of Negative Evaluation Scale (BFNE)**				

Mean (SD)	28.7(5.9)	33.9 (7.6)	0.96	0.006

Range	15–53	18–53		

**Social Phobia Inventory (SPIN)**				

Mean (SD)	18.8 (11.2)	30.9 (7.4)	0.86	<0.001

Range	0–68	0–68		

**Social Interaction Anxiety Scale (SIAS)**				

Mean (SD)	23.8 (12.6)	34.3 (9.8)	0.87	< 0.001

Range	0–53	12–53		

Finally principal component analysis with varimax rotation loaded two factors. The results indicated two distinct factors consisting of straightforward items and reverse-worded questions that jointly accounted for 51.6% of variance observed. The results are shown in Table 4.

**Table 4:** Principle component analysis of the Brief Fear of Negative Evaluation Scale (BFNE)

**Items (item's number)**	**Factor 1**	**Factor 2**
I worry about what other people will think of me even when I know it doesn't make any difference. (1)	**0.70**	0.02

I am frequently afraid of other people noticing my shortcomings. (3)	**0.68**	0.09

I am afraid that others will not approve of me. (5)	**0.71**	0.12

I am afraid that people will find fault with me. (6)	**0.73**	0.11

When I am talking to someone, I worry about what they may be thinking about me. (8)	**0.85**	0.08

I am usually worried about what kind of impression I make. (9)	**0.80**	0.10

Sometimes I think I am too concerned with what other people think of me. (11)	**0.76**	0.07

I often worry that I will say or do the wrong things. (12)	**0.74**	0.02

I am unconcerned even if I know people are forming an unfavorable impression of me. (2)	0.19	**0.64**

I rarely worry about what kind of impression I am making on someone. (4)	0.11	**0.67**

Other people's opinions of me do not bother me. (7)	0.24	**0.42**

If I know someone is judging me, it has little effect on me. (10)	0.07	**0.71**

**Variance contributed by each factor**	34.4	17.2

## Discussion

The BFNE is a well-known instrument for measuring fear of negative evaluation from others and is relevant to the study of human social behavior in general. This study reports data from a validation study of the BFNE in Iran. In general, the findings showed promising results and were comparable with most research findings throughout the world [4,5].

The Iranian version of the BFNE proved to be acceptable to participants and similar to most studies, its reliability as measured by internal consistency and test-retest analysis was found to be satisfactory. Significant correlations were obtained between the BFNE and the SPIN and the SIAS, supporting the convergent validity of the BFNE Scale. This finding is consistent with previous research demonstrating a positive relationship between the BFNE and other measures of social anxiety [7,18]. Weeks et al. [7] found that the BFNE scores correlated to other measures of social phobia such as the SIAS (r = 0.38) and the Social Phobia Scale-SPS (r = 0.35). Carleton et al. reported similar results where they found a significant correlation between the BFNE-II and the SPS (r = 0.60), and the SIAS (r = 0.64) [11].

In support of the discriminant validity of the BFNE, individuals with social phobia scored significantly higher on the scale than non-anxious students. The differences in scores on the BFNE highlight the discriminant ability of the measure for detecting clinically significant levels of social anxiety.

In line with other studies that evaluated the factor structure of the BFNE, factor analysis in the current study supported a two-factor model with factors representing positive and reverse-worded items. As suggested it seems that the reverse-worded factor might be due to the result of students' misunderstanding the double-negative wording in these items. In fact this result show that using reverse-worded items not only might be confused by clinical and community samples, but the educated participants such as university students also might found difficulty in responding to such questions.

This study has several limitations. Perhaps the main concern is that we translated and validated the BFNE scale while evidence suggest that this measure is now out of date and instead the BFNE-II is recommended for measuring social phobia. Secondly, the statistical analysis was limited. For instance, as suggested it would be interesting to carry out ROC analysis. Unfortunately since clinical cut offs of the SIAS, and the SPIN were not established in Iran or a 'gold standard' was not available for the study, we were unable to carry out such analyses.

## Conclusion

This validation study of the Iranian version of BFNE proved that it is an acceptable, reliable and valid measure of social phobia. However, since the scale showed a two-factor structure and this does not confirm to the theoretical basis for the BFNE, thus we suggest the use of the BFNE-II when it becomes available in Iran. The validation study of the BFNE-II is in progress.

## Competing interests

The authors declare that they have no competing interests.

## Authors' contributions

AT wrote the first draft of the manuscript. AT and MM conceptualized and designed the study, coordinated the translation process, collected and analyzed the data. MB contributed to the study design. GHG supervised the study. MS contributed to the data collection. AM analyzed the data further and wrote the final manuscript. All authors read and approved the paper.

## Pre-publication history

The pre-publication history for this paper can be accessed here:


